# Orthohantaviruses: An Overview of the Current Status of Diagnostics and Surveillance

**DOI:** 10.3390/v17050622

**Published:** 2025-04-26

**Authors:** Maria Anele Romeo, Silvia Tofani, Daniele Lapa, Cosmina Mija, Fabrizio Maggi, Maria Teresa Scicluna, Roberto Nardini

**Affiliations:** 1 Laboratory of Virology and Biosafety Laboratories, National Institute for Infectious Diseases Lazzaro Spallanzani—IRCCS, 00149 Rome, Italy; 2 Virology Unit, Istituto Zooprofilattico Sperimentale del Lazio e della Toscana “M. Aleandri”, Via Appia Nuova 1411, 00178 Rome, Italyroberto.nardini@izslt.it (R.N.)

**Keywords:** orthohantaviruses, diagnostics, surveillance

## Abstract

Orthohantavirus infection is a rodent-to-human zoonotic disease with a worldwide distribution, resulting in more than 200,000 cases per year. Human infection leads to two diseases, haemorrhagic fever with renal syndrome and hantavirus cardiopulmonary syndrome, with mortality rates ranging from 1% to 38%. Apart from the data on cases presenting obvious clinical symptoms, the true prevalence is poorly understood, especially in the occupational groups considered to be at risk of exposure. As there is currently no approved therapy or vaccine, surveillance is essential to locate the presumed site of infection following orthohantavirus outbreaks in order to control the spread of infection. To this end, the use of rapid diagnostic tools is essential to rapidly provide data on viral circulation. This review focuses mainly on the available diagnostic methods, both serological and biomolecular, and the surveillance systems used for orthohantaviruses. The information gathered could provide a valid basis for the implementation of further surveillance systems in a country lacking up-to-date data.

## 1. Introduction

Orthohantaviruses (also known as hantaviruses (HTVs)) (order *Bunyavirales*, family *Hantaviridae*, subfamily *Mammantaivirnae*, genus *Orthohantavirus*) [1] are emerging zoonotic viruses that have been classified by the Centers for Disease Control and Prevention (CDC) as an A category pathogen that poses a significant public health burden worldwide [2]. According to data obtained in recent years, an estimated 200,000 people worldwide are infected each year. Although some countries have yet to document cases of human orthohantavirus infection, in part because it can be asymptomatic, the overall number of countries reporting and documenting cases has been increasing [3].

More than 20 orthohantaviruses are known to cause illnesses in humans through rodent transmission [4]. Orthohantaviruses are non-arthropod-borne viruses, and rodents or insectivores are the main natural hosts, which can be persistently infected with little or no pathological consequences [5]. They are detected every year throughout the world and are conveniently divided into two categories based on the region of the epidemic: Old World orthohantaviruses and New World orthohantaviruses [6]. The Old World orthohantavirusones cause haemorrhagic fever with renal syndrome (HFRS) in Europe and Asia [6,7]. However, the most common ones associated with human disease are Hantaan Virus (HTNV), Seoul Virus (SEOV), Puumala Virus (PUUV), and Dobrava-Belgrade Virus (DOBV). SEOV, DOBV, and PUUV were first discovered in South Korea in 1982 [8], Slovenia in 1992 [9], and Finland in 1980 [10], respectively. HTNV and SEOV circulate mainly in Asia, where their main hosts are *Apodemus agrarius* and *Rattus norvegicus*, respectively. DOBV has also been identified in *Apodemus flavicollis*, *A. agrarius*, and *A. ponticus* and is currently circulating in the Balkans, Russia, and Denmark [11,12]. In Europe, DOBV is the most pathogenic to humans, and many lineages of this virus with varying virulence have been identified [13]. The geographical location of an HTV is closely linked to the distribution of reservoirs, with a very close co-evolution between rodents and viruses over millions of years [5,14]. PUUV, for example, is commonly found in bank voles (*Myodes glareolus*), whose range extends from Spain to Siberia and from the Balkans to Northern Scandinavia [15,16]. The migration of bank voles contributes to the spread of PUUV; the movement of infected animals allow the spread of the virus into new areas. When the bank vole rodent population peaks, PUUV infection and reinfection of bank voles occasionally occur, contributing to the genetic diversity of this virus [17]. In addition, contact between different infected rodent groups leads to the exchange of PUUV strains through re-infection and co-infection, resulting in many different PUUV genome variants and the formation of new strains [18]. Eight PUUV genetic lineages have been identified in bank voles.

New World orthohantaviruses cause hantavirus cardiopulmonary syndrome (HCPS) in the Americas. Approximately 300 cases of HCPS are diagnosed each year in this area, mainly in Argentina, Brazil, and Chile, with varying degrees of fatality depending on the viral strain [19]. The most severe forms of HCPS are associated with Sin Nombre virus (SNV), Andes virus (ANDV), Araraquara virus (ARAV), and Juquitiba virus (JUQ), with the first two that possess a fatality rates of 30% and 45%, respectively. Choclo virus (CHOV) and Laguna Negra virus (LANV), on the other hand, have a fatality rate of between 12% and 15% [14,20]. These high mortality rates lead to an inverse correlation between seroprevalence and disease severity; the milder the disease, the higher the seroprevalence [21]. Cases of HCPS occur mainly in spring and summer, when rodent populations increase. In Chile, for example, local increases in *Oligoryzomys longicaudatus* populations during bamboo planting and sowing lead to increased ANDV infections in rodents and humans [22]. For ANDV, person-to-person transmission with a high fatality rate was demonstrated during an outbreak in Argentina in 2018–2019 [23]. ANDV has also been shown to be more resistant to inactivation than PUUV or HNTV [24].

## 2. Orthohantavirus Genome, Replication, and Reassortment

**Figure 1:**
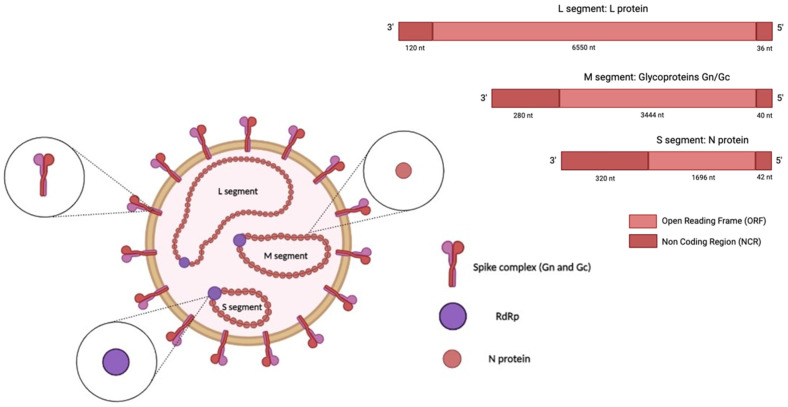
Structure of an orthohantavirus. The HTV genome (-ssRNA) is composed of three RNA segments: small (S), medium (M), and large (L). The S segment encodes the nucleocapsid (N) protein, the medium (M) segment encodes the envelope glycoproteins Gn and Gc, and the L segment encodes the viral RNA-dependent RNA polymerase (RdRp). The image was created on Biorender.com.

Orthohantaviruses are negative, single-stranded RNA viruses with a genome composed of three RNA segments, namely, small (S), medium (M), and large (L), which are 1828, 3650, and 6550 nucleotides (nt) long, respectively (Figure 1) [25,26].

The S segment encodes the nucleocapsid (N) protein; the middle (M) segment encodes a glycoprotein precursor (GPC) that is co-translationally cleaved into two viral envelope glycoproteins, Gn and Gc; and the L segment encodes the viral RNA-dependent RNA polymerase (RdRp) [27]. In addition, the S segment of some orthohantavirus species encodes a non-structural (NS) protein [28]. The N protein is the most abundant viral protein synthesised early after infection [29]. This protein is involved in the intracellular transport and assembly of mature virions and facilitates the attachment of the virus to the host-cell proteins that direct replication [30,31]. When encoded, the NS protein inhibits the interferon-β (IFN-β) promoter, which regulates the interferon response [32]. The glycoproteins Gc and Gn are directly involved in the binding of the cellular receptor and in the entry mechanism; they may also modulate the host immune response [14,30]. Virus entry occurs through different intracellular pathways in a species-dependent manner. The entry route involves clathrin-dependent endocytosis, exhibited in the prototypical HTNV infection [33]; micropinocytosis; and clathrin-independent receptor-mediated endocytosis [34].

RdRp mediates replication and transcription via an incompletely understood mechanism [30]. The replication processes begin with the attachment of the pathogens to the host-cell receptor, followed by membrane fusion, transport, and release into the cytoplasm; the transcription, replication, and translation of the genome; and the assembly and release of the virion [35]. Viral replication occurs in an intermediate compartment called the endoplasmic reticulum–Golgi intermediate compartment (ERGIC) [34]. Several studies suggest that the viral Gn protein interacts with integrin receptors on the surfaces of host cells for binding [36]. In addition, some orthohantaviruses enter host cells as a result of the binding of a currently unknown viral factor to the integrins alphaVβ3 and alphaVβ1, which are heterodimeric transmembrane glycoproteins composed of a and b subunits [35].

The virions are assembled in the Golgi apparatus, and the synthesised virions bud into the Golgi pool [35]. The virions are transported to the cell membrane and released via exocytosis. Some orthohantaviruses can be assembled in the plasma membrane through the fusion of viral vesicles and cell membranes [37].

Vascular endothelial cells and macrophages are the primary sites of orthohantavirus replication [38]. The heterogeneity of these viruses is a consequence of their close co-evolution with their hosts, mainly rodents, but also bats, reptiles, and fish [39]. Orthohantavirus reassortment events were first reported for SNV in 1995, after its discovery [40]. The occurrence of reassortment events is also well documented for the most common European orthohantavirus, PUUV [41]. A study conducted in Finland demonstrated the presence of all six possible segment combinations, evaluating reassortment between two phylogenetic clusters within the same lineage [42]. It has been demonstrated that reassortants with exchanged M segments are better tolerated and likely to be particularly beneficial, while reassortment between S and L segments requires a high degree of genetic compatibility [43].

## 3. Clinical Aspects in Humans and Other Animals

As mentioned above, there are two medically important syndromes, HFRS [44,45,46,47,48,49,50] caused by Old World Orthohantaviruses and HCPS [51,52,53] caused by New World Orthohantavirus infection.

HFRS was discovered in Korea in 1951, at the height of the conflict in the country. In this year, hundreds of U.S. military personnel were hospitalised with fever and oral, nasal, and internal haemorrhages; these cases sometimes led to fatalities related to renal failure and shock [8]. HCPS was identified as the etiologic agent of an outbreak of severe respiratory illness in the Southwestern United States in 1993 [54].

The presentation and severity of infections depend on the type of species involved. Generally, HFRS is characterised by severe lung infections, with coughing and wheezing, associated with renal failure and haemorrhagic manifestations that vary from petechiae to severe internal bleeding [55]. The typical clinical course of HFRS has five phases: febrile, hypotensive, oliguric, diuretic, and convalescent. The earliest stage, the febrile phase, includes symptoms such as fever, pain, and oedema for 3–5 days. The hypotensive phase is characterised by internal bleeding, low blood pressure, thrombocytopenia, and proteinuria. The oliguric phase, in which there is a decrease in urine output, lasts between 3 and 7 days and is characterised by renal dysfunction, electrolyte imbalance, and hypervolemia. The last two phases, the diuretic and convalescent phases, are recovery phases lasting several weeks to months, characterised by progressive improvements in the glomerular filtration rate and renal blood flow [56].

HCPS is characterised by pneumonia and cardiovascular dysfunction with an increased permeability of the microvascular endothelium [57]. Orthohantavirus infections in animals and humans occur mainly in renal and pulmonary endothelial cells (ECs) and macrophages. The pathogenesis of HFRS and HCPS/HPS also involves increased vascular permeability and acute thrombocytopenia [9]. In children, the clinical course of HFRS and HCPS appears to be similar to that in adults. Abdominal pain and vomiting are common in children with a PUUV infection, but the clinical course otherwise seems similar to or milder than that in adults [58,59]. In pregnant women, no differences in the severity of PUUV, DOBV, and/or ANDV infections compared to that in non-pregnant women have been described, but HTNV infections seem to be more severe, causing obstetric or foetal complications in the third trimester [60,61,62]. Miscarriage or preterm labour (before 37 weeks) appears to be due to maternal infection (hypoxemia and hypotension) rather than foetal infection. Intrauterine transmission has not been reported for SNV, ANDV, PUUV, DOBV, or SEOV; but two cases of ANDV infection were PCR-positive in mothers breastfeeding infants, thus demonstrating transmission of the virus [63,64].

The viruses responsible for both diseases’ forms are transmitted through inhalation of aerosols or dust particles containing rodent-contaminated excreta (mice, rats, shrews, and voles), with no arthropod vectors involved, unlike with other viruses of this family [9,55]. Additionally, it has been demonstrated that ANDV, a New World species, can be transmitted person-to-person [57]. The most common serotypes causing HFRS are HTNV, DOBV, SEOV, and PUUV; rodents are asymptomatic reservoirs of the viruses. The rate of the spread of infection to humans is over 100,000 cases per year, with an outcome that is usually self-limiting [65]. In the Americas, where SNV and ANDV are present, these viruses may cause orthohantavirus pulmonary syndrome, with case-fatality rates of approximately 40%. HNTV in Asia, PUUV and DOBV in Europe, and SEOV worldwide may cause HFRS with varying degrees of severity, with an average mortality rate of 12% [66,67]. PUUV, the most evident and widely occurring orthohantavirus in Europe, is transmitted principally by the bank vole, causing a mild form of HFRS, which is characterised by acute kidney failure that usually clears up spontaneously within days, while the concomitant renal failure can be severe [66]. DOBV is transmitted by the yellow-necked field mouse (*Apodemus flavicollis*), and it causes a more severe form of HFRS [16,68]. In addition, the discovery of soricidborne orthohantaviruses suggested that moles (order *Eulipotyphla*, family *Talpidae*) might also harbour these orthohantaviruses, together with several talpid-borne orthohantaviruses that have been found in Europe, Asia, and North America [69].

## 4. Epidemiology

The 1993 outbreak of severe respiratory illness in the Four Corners region (the area where Arizona, Colorado, New Mexico, and Utah meet) led to orthohantavirus disease surveillance in the United States. Afterwards, in 1995, Orthohantavirus pulmonary syndrome (HPS) became a national notifiable disease. Today, the National Notifiable Disease Surveillance System reports that patients with laboratory-confirmed evidence of orthohantavirus infection often present with a fever [70]. In 2014, both HPS and non-pulmonary orthohantavirus infections were included in the national report regarding orthohantaviruses released by the Council of State and Territorial Epidemiologists. From 1993 to the end of 2022, 864 cases of orthohantaviruses disease were reported in the United States, including HPS and non-pulmonary orthohantavirus infection [71].

In Europe, data on communicable diseases are collected by the European Surveillance System (TESSy). The latest report on orthohantavirus was issued in 2021. In 2020, 28 countries reported 1647 cases, 1643 (99.8%) of which were classified as confirmed, and the remaining 4 (0.2%) cases were reported as probable. Ten countries reported zero cases. Two countries (Finland and Germany) accounted for 85% of all the reported cases, with Finland alone accounting for 71% of all cases. The same pattern was observed from 2016 to 2020, when Finland and Germany accounted for over 74% of annual cases [72]. Today, Finland still has the highest orthohantavirus disease incidence globally, with 1000–3300 human PUUV infections diagnosed annually [73].

The most frequently identified pathogen was PUUV (98.3%), while HTNV was identified in 14 cases (13 in Slovakia and 1 in Slovenia) and DOBV in 7 cases. The disease mostly affects adults over 25 years of age. There is an increase in cases during the November–December period in countries in Northern Europe because humans make contact with infected rodents in the countryside more frequently in this period. The same is observed during the summer due to an increased exposure of urban dwellers during their summer holidays [68,74].

The orthohantaviruses of the Old and New World were first discovered following two major outbreaks. The first was during the Korean War (spanning from 1950 to 1953), with over 3000 United Nations troops falling ill with Korean haemorrhagic fever or HFRS [14]. The agent remained unknown until 1978, when a new virus, HTNV, named after the Hantaan river, was isolated in its rodent host, the striped field mouse (*Apodemus agrarius*). A retrospective analysis of sera collected from soldiers during the Korean conflict confirmed that KHF was caused by HTNV [75].

**Figure 2:**
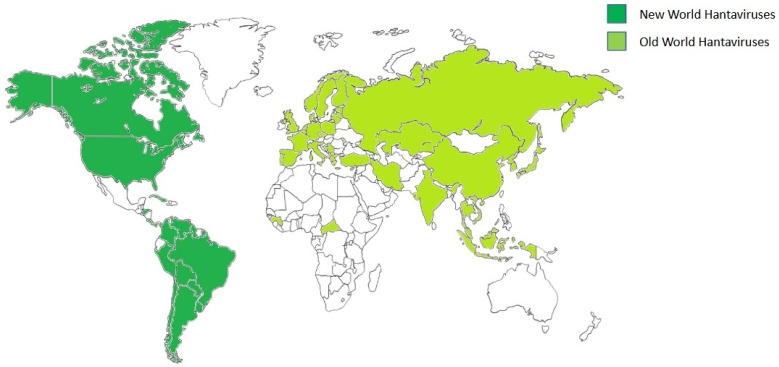
Geographical distribution of orthohantaviruses. Global geographic distribution of Old World orthohantaviruses (light green) and New World orthohantaviruses (dark green) affecting humans. The image was created on Biorender.com.

The second outbreak of disease occurred in the Four Corners region of the United States in 1993; this form of the disease is now called HPS or HCPS [14]. There were other cases among soldiers from Croatia during the 1987–2001 period, and the causative agents were Puumala and Dobrava viruses [75]. An estimated 28 Orthohantaviruses causing diseases in humans have been identified around the world, with 1000 HCPS cases and more than 100,000 HFRS cases reported [57] (Figure 2).

No human cases of orthohantavirus infection have been officially reported in Italy in the last decade in the native population, despite its proximity to endemic countries and the presence of both the wild yellow-necked mouse, the black-striped field mouse, and the bank vole. Only eight cases of orthohantavirus disease have been described, mostly in tourists or transboundary workers and possibly related to an infection contracted abroad [39]. Spain reported only one case in 2017 [74].

The remaining European countries, such as Cyprus, Iceland, Ireland, Lithuania, Malta, and Portugal, have not reported any cases in the last decade.

## 5. Diagnosis

### 5.1. Serological Diagnosis

**Figure 3:**
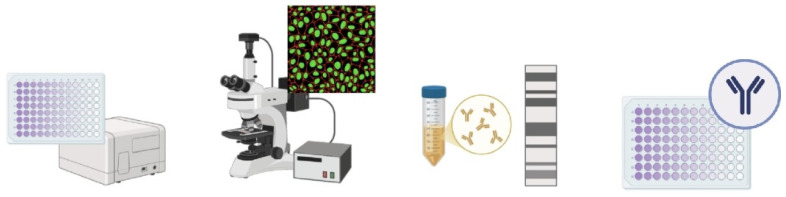
Schematic representation of serological methods for diagnosing HTV. The image was created on Biorender.com.

Serological tests are the key test for orthohantavirus diagnosis according to worldwide guidelines [70,76]. Serological tests, carried out using various techniques, allow the detection of immunoglobulin M (IgM) and immunoglobulin G (IgG) antibodies produced to combat the virus in a patient’s serum sample. The detection of IgM indicates recent exposure to the virus and an acute phase of the disease, whereas the presence of IgG indicates a convalescent phase of the disease. The IgG antibody may have lifelong persistence and could be useful in monitoring the evolution of the disease in a population [77], whereas IgM antibodies indicate recent infection but may take up to 2 weeks to appear [5]. The principal serological methods are enzyme-linked immunosorbent assays (ELISAs), immunofluorescence assays (IFAs), immunoblot assays (IBAs), the plaque reduction neutralisation test (PRNT), and the focus reduction neutralisation test (FRNT) (Figure 3).

#### 5.1.1. ELISA

The ELISA, which has been widely used since the 1990s, is an automated and very simple test that allows easy differentiation between IgG and IgM antibodies. This test usually involves the use of either native purified antigens, recombinant nucleocapsid proteins (rNp), or even truncated rNp as antigens [5]. There are currently several commercial kits that have shown comparable performance. The advantage of this test is that it can be used with different sample types (e.g., plasma and serum); however, this test is not able to distinguish the specific strain of the virus responsible for the infection. In addition, as determined by an external quality assessment (EQA) performed in Europe, this test does not show high performance regarding both specificity and sensitivity characteristics, particularly for the diagnosis of IgM in samples with highly diluted positive samples [78].

#### 5.1.2. Immunofluorescence Assay

It has been shown that the only method that does not give false-negative results is a combination of ELISA and IFA [78], the latter being the most widely used in Europe. This test is based on the reactivity of the serum with orthohantavirus-infected cells fixed on microscope slides; again, IgM and IgG can be easily distinguished. In-house slides are not widely used because such practices require biosafety level 3 (BSL-3) laboratories, but commercial kits are available. The commercial kits entail the use of a biochip coated with uninfected cells or cells infected with different strains of orthohantavirus. The use of this biochip additionally allows the identification of the specific strain of HNTV, but there have been cases of cross-reactivity between viruses [79].

#### 5.1.3. Immunoblot Assay

IgM and IgG can also be distinguished by the IBA assay, which EQA has shown to have better performance than the ELISA and IFA assays [78]. IBA could be useful as a rapid test or as a confirmation test. The commercially available kits contain as antigens the complete nucleocapsids of Puumala and Hantaan viruses or a recombinant N-terminus of the nucleocapsid antigen of Dobrava, Seoul, Puumala, and Hantaan viruses together with control bands that react with antibodies in a patient’s serum [79].

#### 5.1.4. Neutralisation Test

The PRNT and FRNT assays allow the measurement of antibody titres in patient serum collected during the acute phase of a disease. Neutralising antibody titres are determined by a patient’s serum dilution, followed by using immunoperoxidase staining and immunocolorimetric approaches to detect IgG, respectively. The antibody titres are expressed as the reciprocal of the highest serum dilution that results in an 80% or greater reduction in the number of viral plaques and/or foci [8,77]. A stain must be used due to the lack of a cytopathic effect associated with HNTV infection in Vero E6 cells. The advantage of FRNT compared to PRNT is that it allows visualisation of viral foci via immunostaining after only 2 or 3 days of infection. The disadvantages of these tests are that they can only be performed in BSL-3 laboratories, as they require the use of a live virus via a cell culturing technique; are time consuming; and require highly specialised personnel [77]. To this end, viable alternatives such as the microneutralisation test (MNT) and the pseudoparticle neutralisation test (PPNT), which could be used as simple and rapid alternatives, were investigated. It was shown that the MNT assay is more specific, while the PPNT assay is more sensitive, than other assays for the determination of neutralising antibodies in HNTV infection [80].

### 5.2. Molecular Diagnosis and Sequencing

**Figure 4:**
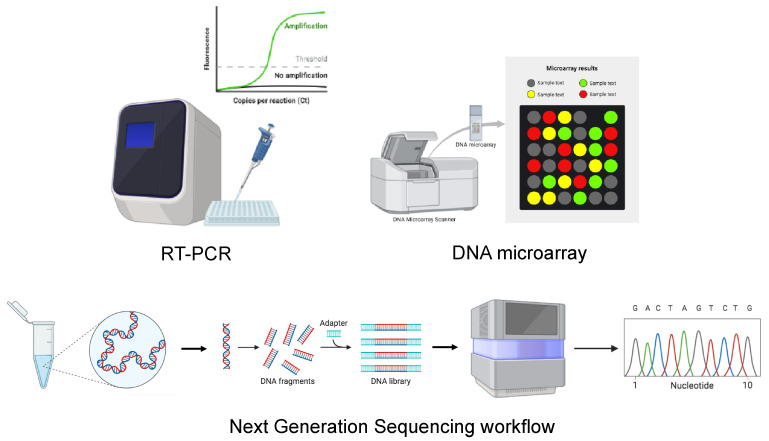
Schematic representation of molecular methods for the diagnosis of HTV. The image was created on Biorender.com.

The intricate taxonomy and sequence diversity of orthohantaviruses make the detection of hantaviral RNA via RT-PCR very complex, even at the low viral loads in clinical samples [81]. For this reason, the diagnosis of orthohantaviruses is currently based on serological assays, while molecular techniques, which can be performed using either blood or plasma samples, are used only as confirmatory tests. The major advantage of RT-PCR is its ability to detect viruses in the early stages of a disease, allowing early identification of infection with a high risk of fatal disease [77]. In addition, to identify new orthohantavirus genetic variants, a DNA microarray and next-generation sequencing (NGS) techniques can also be used (Figure 4).

#### 5.2.1. RT-PCR

Currently, only two RT-PCR commercial kits have been developed by Altona Diagnostics; these commercial RT-PCR kits are specific for the detection of orthohantavirus species that cause HFRS or HPS [82,83]. There are no data on the gene target used or specificity and sensitivity for these tests, which have been deemed to be for “Research Use Only” [83]. The in-house PCR approach, usually developed with reference to a specific orthohantavirus strain, is also used, exhibiting excellent results in terms of sensitivity and specificity. In Europe, the most used approach was described by Kramski et al. [81]. Kramski et al.’s group, using a gene target, namely, a highly conserved region within the S-segment of a specific orthohantavirus genome, developed three different real-time RT-PCR assays for the specific detection of the European orthohantaviruses DOBV, PUUV, and TULV and two real-time RT-PCR assays for the simultaneous detection of the Asian species HNTV and SEOV and the American species ANDV and SNV. The real-time RT-PCR assays were specific for the desired orthohantavirus species even in concentrations close to the detection limit of 10 copies per reaction, with a high level of reproducibility. Kramski et al.’s approach is currently the most widely used test worldwide for the detection of both Old World and New World orthohantaviruses [1,84,85,86]. Another in-house PCR approach was developed by Atichou et al. for the detection of four Old World orthohantaviruses (DOBV, HTNV, PUUV, and SEOV) [87]. The primers and probes were designed after multiple aligning of S segment nucleotide sequences from different strains or isolates of HTNV, DOBV, PUUV, and SEOV. The sensitivity of this approach was 98%, 96%, 92%, and 94%, respectively, with the specificity for the DOBV, HTNV, and SEOV assays being 100% and that of the PUUV assay being 98%. The detection limits, measured as plaque-forming units (p.f.u.) in this paper, were 25, 25, 25, and 12.5 p.f.u. for the DOBV, HTNV, PUUV, and SEOV assays, respectively. Many of the in-house approaches use the S-segment as a gene target, and this is also the case for the multiplex approach [88], with good results in terms of sensitivity and specificity. The use of the L-target for the development of a nested in-house PCR was described by Klempa et al. for murine screening in Guinea [89]. This approach is currently used by some European laboratories, with unsuccessful results in terms of orthohantavirus detection in human samples, as demonstrated in the EQA analysis [90]. Table 1 summarises the RT-PCR methods available in the literature.

#### 5.2.2. DNA Microarray

DNA microarrays can also be used for HNTV analysis for clinical and epidemiological purposes [96]. DNA microarrays enable high-throughput screening of DNA fragments and are powerful tools for identifying new genetic variants of emerging viruses. The third generation of PathogenID v3.0 uses the S segment encoding the N protein as a tiled sequence, making it very efficient for discriminating variants within a species. This technique is advantageous because it allows one to explore the genetic space of orthohantaviruses and accurately identify local variants present in the infected tissue or cell supernatant [97].

#### 5.2.3. Viral Sequencing

Analysis of partial viral genome sequences or the whole-genome sequencing of infectious agents allows the identification, characterisation, and epidemiological surveillance of orthohantaviruses. During an outbreak, understanding the dynamics of transmission, building a database of viral genome sequences, and evaluating the epidemiological association between patients and sources of infection can be useful for the development of a timely response when formulating outbreak control strategies [56,98,99]. Before the improvement of NGS techniques, partial genome sequences were used to monitor the emergence of HNTV in the Republic of Korea [100,101], analyse serum samples from US soldiers during an outbreak in 2005 [102], and analyse and diagnose HNTV infection in 31 HFRS cases in Germany [103]. However, the analysis of partial genome sequences does not allow a full examination of the genomic variants of a virus, leading to a bias in the analysis of the phylogenetic position of the virus [100]. The introduction of NGS, in turn enabling whole-genome sequencing, has allowed improvements in active viral surveillance and led to a better understanding of characteristics and transmission dynamics during outbreaks. NGS technology has been used and is being used for viral genomic epidemiology regarding human and natural reservoir specimens [104] (Figure 4). It has been demonstrated that this technology enabled the detection of human-to-human or animal-to-human transmission during the Ebola Virus (EBOV) outbreak in West Africa in 2014 [105], the Lassa Virus (LASV) outbreak in Nigeria in 2018 [106], and the Zika Virus (ZIKV) outbreak in the United States in 2016 [107]. For HNTV outbreaks, NGS is mainly used to perform phylogeographic analyses comparing patients with HFRS with HNTV-positive rodents captured at suspected patient infection sites to confirm the transmission route [100,108,109,110] or to perform a genome-wide association study (GWAS) to identify genetic variants associated with infection status in a reservoir host [111]. An MiSeq benchtop sequencer (Illumina) or MinION (Oxford Nanopore Technologies) can be used as an NGS platform, yielding comparable results [110,112]. The increased speed and ease of use of the Nanopore platform highlight its potential for use as a point-of-care test for suspected patients during an orthohantavirus outbreak. A major limitation of whole-genome sequencing for viral sequence identification is the extremely low copy numbers of viral genomes in clinical samples. To this end, several NGS approaches have been developed, allowing enrichment of target genomes. Among the NGS approaches, single-primer amplification (SISPA), specific oligonucleotide probe-mediated enrichment of target viral nucleic acids, and amplicon-based NGS are the most widely used [108,113].

### 5.3. Virus Isolation in Cell Culture

The isolation of HNTV from human material is usually performed using Vero E6 cells, and it is a very difficult process [114]. Due to the lack of cytopathic effects in an vitro infection, virus isolation is performed through three blind passages in new monolayers of Vero E6 cells, followed by IFA orthohantavirus antigen testing using a seropositive serum sample [115]. Due to its difficulty and the need for specially trained personnel and BSL-3 conditions, this method is rarely used.

### 5.4. Immunohistochemistry

Immunohistochemistry using ELISA and IF can also be used to detect HNTV in infected organs. The main disadvantages of these techniques are that they require elaborate preparations and are mostly used post-mortem [116].

## 6. HTV Surveillance in Humans and Animals

**Figure 5:**
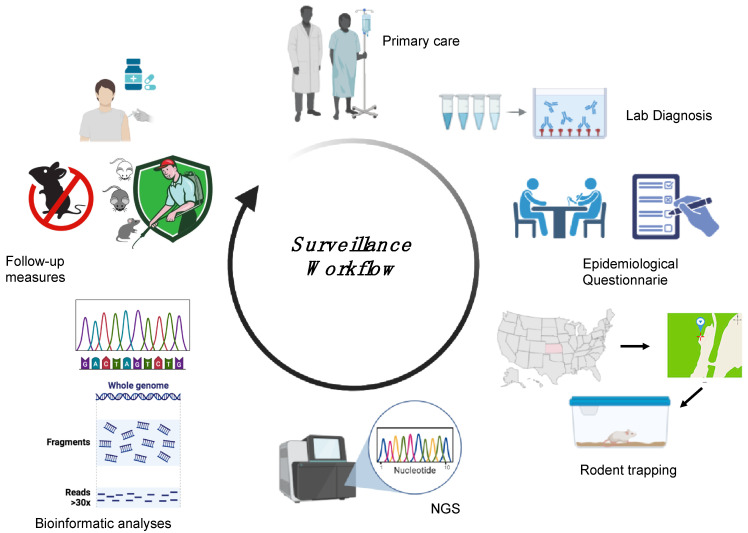
Surveillance workflow followed during HTV outbreaks. The patient is admitted to a hospital with suspected HFRS/HCPS. Following a positive laboratory diagnosis, an epidemiological questionnaire is provided to the patient, allowing the identification of a suspected site of contact between the patient and the rodent. Capturing the rodents at the suspected site, followed by performing HTV genomic analysis of rodents and humans, allows a phylogeographic link to be established between the patient and rodent-derived HTV strains, and this link can be used to implement follow-up measures in the specific area to prevent new infections. The image was created on Biorender.com.

Surveillance is essential to locate the presumed site of infection following HFRS or HCPS outbreaks and thus determine the level of spread and define categories of risk, yet very little research is available describing the surveillance approaches applied in different countries. Each surveillance activity relies on a precise definition of suspected and confirmed cases. For orthohantaviruses, the World Health Organization (WHO) defines a suspected case as either “a person who presents with a febrile illness (Fever > 38.5 °C [103 °F] oral) with an acute respiratory distress syndrome requiring supplemental oxygen and bilateral diffuse infiltrates developed within 72 h of hospitalisation in a previously healthy person” or “unexplained illness resulting in death plus an autopsy examination demonstrating noncardiogenic pulmonary oedema without an identifiable specific cause of death” and a confirmed case as “a suspected case laboratory confirmed with: the presence of orthohantavirus-specific IgM antibodies or a 4-fold or greater increase in IgG antibody titres or orthohantavirus-specific IgG seroconversion; a positive reverse transcriptase-polymerase chain reaction (RT-PCR) results for orthohantavirus RNA; positive immunohistochemical results for orthohantavirus antigens” [117]. A specific method of confirmation among those available has not yet been identified, so the Pan American Health Organization, in 2024, organised a meeting of international collaboration on orthohantavirus surveillance, which included among its topics a review of laboratory protocols, equipment, and strategies, with an emphasis on molecular techniques [118]. Surveillance includes both animals and humans and gathers information on the source of infection and the specific reservoir species. HTV surveillance involves five different steps: laboratory diagnosis, an epidemiological questionnaire, reservoir capture, sequencing analysis, and follow-up measures (Figure 5) [56,100]. Following a confirmed diagnosis of HFRS or HCPS, an epidemiological investigation is essential to identify a suspected site of contact between the patient and the rodent. Capturing rodents at the suspected site of infection, followed by conducting an HTV genomic analysis of the rodents, allows comparison of the HTV genome found in the captured rodents with that in the infected human cases. This workflow will allow the establishment of a phylogeographic link between patient and rodent-derived HTV strains with which to implement follow-up measures in the specific area in order to reduce the incidence of HTV infection [56,100,119]. Surveillance has proven to be crucial for the identification of new HTV strains and specific reservoir organisms, such as in the identification of SNV [119]. The implementation of surveillance also allows rapid communication of confirmed cases to the European Centre for Disease Prevention and Control (ECDC) and CDC in Europe and the United States, respectively. Data are transmitted directly from national health authorities to surveillance centres to ensure effective and timely surveillance of orthohantavirus cases in each country through the development of annual reports [74].

## 7. Discussion and Conclusions

Orthohantaviruses cause severe zoonotic diseases in humans, and their reservoirs are rodents, both wild and synanthropic. A reliable diagnosis, together with a surveillance system, is crucial for monitoring the presence of these viruses and implementing preventive measures in a human public health setting. Diagnostic methods for orthohantaviruses typically include serological assays (such as ELISA) used to detect specific antibodies and PCR for viral RNA detection. Commercially, very few assays are available, and, in the literature, while some diagnostic protocols are present, they are mostly employed for research, and a uniquely approved method has not yet been adopted for both human and veterinary diagnosis [117,120]. The optimal selection of a method of diagnosis and a diagnostic algorithm could result in a more reliable dataset that can be shared within the scientific community and aid political decision-making.

According to Escadafal et al., who used a panel of HTV-negative and PUUM- and DOB-positive sera for either or both IgG and IgM, immunoblotting appears to be slightly more sensitive than IFA and ELISA, serological assay performance is comparable but varies between laboratories even when using the same assay, and the quality of results is much more influenced by the proper standardisation of methods [78].

Regarding molecular methods, as shown in Table 1, shared data on sensitivity and specificity are lacking, as are structured comparison studies, so no conclusions can be drawn.

Integrated surveillance systems play a crucial role in monitoring orthohantavirus activity in rodent populations and human cases, facilitating early detection and responses to outbreaks. Such systems can incorporate environmental monitoring, serological surveys of wildlife, and epidemiological data to predict potential spillover events. Orthohantaviruses are zoonotic pathogens influenced by climate change, which impacts their rodent reservoirs and transmission dynamics, increasing risks in regions previously considered low-risk. Public health authorities, therefore, must also incorporate climate data into their orthohantavirus surveillance and response strategies to adequately prepare for and mitigate the impacts of climate change. The employment of these methods not only aids in an immediate clinical diagnosis but also enables health authorities to assess the geographical distribution of orthohantaviruses, update public health strategies, and raise awareness about preventive measures among at-risk populations in rural and peri-urban areas, particularly in high-risk regions or in regions in which the viruses have been recently detected [55].

## Figures and Tables

**Table 1:** Biomolecular methods available in the literature for screening orthohantaviruses. ANDV: Andes virus; DOBV: Dobrava virus; HTNV: Hantaan virus; PUUV: Puumala virus; ROBV: Robina virus; SAAV: Saaremaa virus; SEOV: Seoul virus; SNV: Sin Nombre Virus; TOPV: Topografov virus; TULV: Tula virus. NA: not available.

Author	Specificity	Sensitivity	Sample Size	Reference Assay	Biological Matrix	Target Gene	Virus(es) Detected	Test Type	TEST
[91]	NA	NA	3	Rapid peroxidase enzyme-linked immunosorbent assay (PAGEIA)	Blood samples	Small (S) and medium (M) segments	SNV	Homemade one-step RT-PCR nested method	SNV-MH strains 1, 2, and 3 RT-PCR
[91]	NA	95–103%	3	Rapid peroxidase enzyme-linked immunosorbent assay (PAGEIA)	Blood samples	Small (S) segment	SNV	qRT–PCR with fluorescent signal	SNV Quantitative Real time RT-PCR
[92]	No cross-reactivity with Hendra virus genotype 1, Hendra virus genotype 2, ABLV, Kunjin virus, Murray Valley encephalitis virus, Ross River virus, or Japanese encephalitis virus	NA	NA	NA	Brain tissue	Small (S) segment	ROBV	qRT–PCR with fluorescent signal	Quantitative Real time RT-PCR
[92]	NA	NA	NA	NA	Brain tissue	Small (S) segment	ROBV	Homemade one-step RT-PCR nested method	nested RT-PCR
[81]		10 copies	NA	NA	NA		DOBV, PUUV, TULV and HTNV, SEOV, and ANDV and SNV	Homemade five-different-qRT-PCR method	5 real-time RT-PCR assays
[93]	NA	NA	NA	NA	Lung tissues	Small (S) segment	HNTV and SEOV	Homemade one-step RT-PCR nested method	nested RT-PCR
[94]	NA	NA	20	Immunoblotting	Peripheral blood mononuclear cells and lung tissues	Entire S segment and partial M segment	PUUV	Homemade one-step RT-PCR nested method followed by Sanger sequencing	nested RT-PCR
[86]	DOBV, SAAV, HTNV, TULV, and TOPV	3.3 copies per reaction	288	Immunofluorescence assay	Serum samples	Small (S) segment	PUUV	Homemade real-time RT-PCR method modified from Plyusnin A et al.’s one-step RT-PCR method	real-time RT-PCR and nested RT-PCR
[91]	NA	NA	7	Rapid peroxidase enzyme-linked immunosorbent assay (PAGEIA)	Blood samples from deer mice	Partial S segment and partial M segment	SNV	Homemade one-step RT-PCR nested method and homemade real-time RT-PCR method	real-time RT-PCR and nested RT-PCR
[12]	NA	NA	612	NA	Blood samples	Highly conserved L segment	Members of the genus Orthohantavirus	Homemade nested RT-PCR assay	nested RT-PCR
[95]						Partial S segment	Members of the genus Orthohantavirus	Five different RT-PCRs analysed via restriction endonuclease digestion	RT-PCR

## Data Availability

No new data were created or analysed in this study. Data sharing is not applicable to this article.

## Abbreviations

The following abbreviations are used in this manuscript:

ANDV	Andes virus
ARAV	Araraquara virus
BSL-3	biosafety level 3
CDC	Centers for Disease Control and Prevention
CHOV	Choclo virus
DOBV	Dobrava-Belgrade Virus
EBOV	Ebola Virus
ECDC	European Centre for Disease Prevention and Control
ELISA	enzyme-linked immunosorbent assay
EQA	external quality assessment
ERGIC	endoplasmic reticulum-Golgi intermediate compartment
FRNT	focus reduction neutralisation test
GPC	glycoprotein precursor
GWAS	genome wide association study
HCPS	hantavirus cardio-pulmonary syndrome
HFRS	haemorrhagic fever with renal syndrome
HPS	hantavirus pulmonary syndrome
HTNV	Hantaan Virus
HTVs	hantaviruses
IBA	immunoblot assay
IFA	immunofluorescence assay
IFN	interferon
JUQ	Juquitiba virus
LANV	Laguna Negra virus
LASV	Lassa Virus
MNT	microneutralisation test
NGS	next-generation sequencing
NS	non-structural
p.f.u.	plaque-forming units
PPNT	pseudoparticle neutralisation test
PRNT	plaque reduction neutralisation test
PUUV	Puumala Virus
RdRp	RNA-dependent RNA polymerase
rNp	recombinant nucleocapsid proteins
SEOV	Seoul Virus
SISPA	single-primer amplification
SNV	Sin Nombre virus
TESSy	European Surveillance System
WHO	World Health Organization
ZIKV	Zika Virus

## References

[1] Goodfellow S.M., Nofchissey R.A., Ye C., Dunnum J.L., Cook J.A., Bradfute S.B. (2022). Use of a Novel Detection Tool to Survey Orthohantaviruses in Wild-Caught Rodent Populations. Viruses.

[2] Hussein I.T.M., Haseeb A., Haque A., Mir M.A. (2011). Recent Advances in Hantavirus Molecular Biology and Disease. Advances in Applied Microbiology.

[3] Watson D.C., Sargianou M., Papa A., Chra P., Starakis I., Panos G. (2014). Epidemiology of Hantavirus Infections in Humans: A Comprehensive, Global Overview. Crit. Rev. Microbiol..

[4] Noack D., Goeijenbier M., Reusken C.B.E.M., Koopmans M.P.G., Rockx B.H.G. (2020). Orthohantavirus Pathogenesis and Cell Tropism. Front. Cell. Infect. Microbiol..

[5] Clement J., Maes P., Van Ranst M. (2006). Hantaviruses in the Old and New World. Perspectives in Medical Virology.

[6] Chen R.-X., Gong H.-Y., Wang X., Sun M.-H., Ji Y.-F., Tan S.-M., Chen J.-M., Shao J.-W., Liao M. (2023). Zoonotic Hantaviridae with Global Public Health Significance. Viruses.

[7] Tkachenko E.A., Morozov V.G., Dzagurova T.K., Yunicheva Y.V., Pilikova O.M., Zavora D.L., Ishmukhametov A.A., Gorodin V.N., Bakhtina V.A., Zagidullin I.M. (2016). Etiologic and Clinical Epidemiological Features of Hemorrhagic Fever with Renal Syndrome (HFRS) in the Krasnodar Krai. Epidemiol. Infect. Dis..

[8] Lee H.W., Baek L.J., Johnson K.M. (1982). Isolation of Hantaan Virus, the Etiologic Agent of Korean Hemorrhagic Fever, from Wild Urban Rats. J. Infect. Dis..

[9] Avsic-Zupanc T., Xiao S., Stojanovic R., Gligic A., Van Der Groen G., Leduc J.W. (1992). Characterization of Dobrava Virus: A Hantavirus from Slovenia, Yugoslavia. J. Med. Virol..

[10] Brummer-Korvenkontio M., Vaheri A., Hovi T., Von Bonsdorff C.-H., Vuorimies J., Manni T., Penttinen K., Oker-Blom N., Lahdevirta J. (1980). Nephropathia Epidemica: Detection of Antigen in Bank Voles and Serologic Diagnosis of Human Infection. J. Infect. Dis..

[11] Chandy S., Mathai D. (2017). Globally Emerging Hantaviruses: An Overview. Indian J. Med. Microbiol..

[12] Klempa B., Tkachenko E.A., Dzagurova T.K., Yunicheva Y.V., Morozov V.G., Okulova N.M., Slyusareva G.P., Smirnov A., Kruger D.H. (2008). Hemorrhagic Fever with Renal Syndrome Caused by 2 Lineages of Dobrava Hantavirus, Russia1. Emerg. Infect. Dis..

[13] Kolodziej M., Melgies A., Joniec-Wiechetek J., Michalski A., Nowakowska A., Pitucha G., Niemcewicz M. (2018). First Molecular Characterization of Dobrava-Belgrade Virus Found in Apodemus Flavicollis in Poland. Ann. Agric. Environ. Med..

[14] Jonsson C.B., Figueiredo L.T.M., Vapalahti O. (2010). A Global Perspective on Hantavirus Ecology, Epidemiology, and Disease. Clin. Microbiol. Rev..

[15] Madrières S., Tatard C., Murri S., Vulin J., Galan M., Piry S., Pulido C., Loiseau A., Artige E., Benoit L. (2020). How Bank Vole-PUUV Interactions Influence the Eco-Evolutionary Processes Driving Nephropathia Epidemica Epidemiology—An Experimental and Genomic Approach. Pathogens.

[16] Cosseddu G.M., Sozio G., Valleriani F., Di Gennaro A., Pascucci I., Gavaudan S., Marianneau P., Monaco F. (2017). Serological Survey of Hantavirus and Flavivirus Among Wild Rodents in Central Italy. Vector-Borne Zoonotic Dis..

[17] Voutilainen L., Kallio E.R., Niemimaa J., Vapalahti O., Henttonen H. (2016). Temporal Dynamics of Puumala Hantavirus Infection in Cyclic Populations of Bank Voles. Sci. Rep..

[18] Kabwe E., Davidyuk Y., Shamsutdinov A., Garanina E., Martynova E., Kitaeva K., Malisheni M., Isaeva G., Savitskaya T., Urbanowicz R.A. (2020). Orthohantaviruses, Emerging Zoonotic Pathogens. Pathogens.

[19] Vial P.A., Ferrés M., Vial C., Klingström J., Ahlm C., López R., Le Corre N., Mertz G.J. (2023). Hantavirus in Humans: A Review of Clinical Aspects and Management. Lancet Infect. Dis..

[20] Armien B., Pascale J.M., Muñoz C., Mariñas J., Núñez H., Herrera M., Trujillo J., Sánchez D., Mendoza Y., Hjelle B. (2013). Hantavirus Fever without Pulmonary Syndrome in Panama. Am. Soc. Trop. Med. Hyg..

[21] Ferrer J.F., Jonsson C.B., Esteban E., Galligan D., Basombrio M.A., Peralta-Ramos M., Bharadwaj M., Torrez-Martinez N., Callahan J., Segovia A. (1998). High Prevalence of Hantavirus Infection in Indian Communities of the Paraguayan and Argentinean Gran Chaco. Am. J. Trop. Med. Hyg..

[22] Jaksic F.M., Lima M. (2003). Myths and Facts on Ratadas: Bamboo Blooms, Rainfall Peaks and Rodent Outbreaks in South America. Austral Ecol..

[23] Martínez V.P., Di Paola N., Alonso D.O., Pérez-Sautu U., Bellomo C.M., Iglesias A.A., Coelho R.M., López B., Periolo N., Larson P.A. (2020). “Super-Spreaders” and Person-to-Person Transmission of Andes Virus in Argentina. N. Engl. J. Med..

[24] Hardestam J., Lundkvist Å., Klingström J. (2009). Sensitivity of Andes Hantavirus to Antiviral Effect of Human Saliva. Emerg. Infect. Dis..

[25] Schmaljohn C.S., Hasty S.E., Harrison S.A., Dalrymple J.M. (1983). Characterization of Hantaan Virions, the Prototype Virus of Hemorrhagic Fever with Renal Syndrome. J. Infect. Dis..

[26] Kukkonen S.K.J., Vaheri A., Plyusnin A. (2005). L Protein, the RNA-Dependent RNA Polymerase of Hantaviruses. Arch. Virol..

[27] Muyangwa M., Martynova E.V., Khaiboullina S.F., Morzunov S.P., Rizvanov A.A. (2015). Hantaviral Proteins: Structure, Functions, and Role in Hantavirus Infection. Front. Microbiol..

[28] Vera-Otarola J., Solis L., Soto-Rifo R., Ricci E.P., Pino K., Tischler N.D., Ohlmann T., Darlix J.-L., López-Lastra M. (2012). The Andes Hantavirus NSs Protein Is Expressed from the Viral Small mRNA by a Leaky Scanning Mechanism. J. Virol..

[29] Davidyuk Y., Shamsutdinov A., Kabwe E., Ismagilova R., Martynova E., Belyaev A., Shuralev E., Trifonov V., Savitskaya T., Isaeva G. (2020). Prevalence of the Puumala Orthohantavirus Strains in the Pre-Kama Area of the Republic of Tatarstan, Russia. Pathogens.

[30] Khaiboullina S., Morzunov S., St (2005). Jeor, S. Hantaviruses: Molecular Biology, Evolution and Pathogenesis. Curr. Mol. Med..

[31] Flick K., Hooper J.W., Schmaljohn C.S., Pettersson R.F., Feldmann H., Flick R. (2003). Rescue of Hantaan Virus Minigenomes. Virology.

[32] Jääskeläinen K.M., Kaukinen P., Minskaya E.S., Plyusnina A., Vapalahti O., Elliott R.M., Weber F., Vaheri A., Plyusnin A. (2007). Tula and Puumala Hantavirus NSs ORFs Are Functional and the Products Inhibit Activation of the Interferon-beta Promoter. J. Med. Virol..

[33] Jin M., Park J., Lee S., Park B., Shin J., Song K.-J., Ahn T.-I., Hwang S.-Y., Ahn B.-Y., Ahn K. (2002). Hantaan Virus Enters Cells by Clathrin-Dependent Receptor-Mediated Endocytosis. Virology.

[34] Ramanathan H.N., Chung D.-H., Plane S.J., Sztul E., Chu Y., Guttieri M.C., McDowell M., Ali G., Jonsson C.B. (2007). Dynein-Dependent Transport of the Hantaan Virus Nucleocapsid Protein to the Endoplasmic Reticulum-Golgi Intermediate Compartment. J. Virol..

[35] Mittler E., Dieterle M.E., Kleinfelter L.M., Slough M.M., Chandran K., Jangra R.K. (2019). Hantavirus Entry: Perspectives and Recent Advances. Advances in Virus Research.

[36] Mir M.A., Panganiban A.T. (2010). The Triplet Repeats of the Sin Nombre Hantavirus 5′ Untranslated Region Are Sufficient in *Cis* for Nucleocapsid-Mediated Translation Initiation. J. Virol..

[37] Meier K., Thorkelsson S.R., Quemin E.R.J., Rosenthal M. (2023). Correction: Meier et al. Hantavirus Replication Cycle—An Updated Structural Virology Perspective. *Viruses* 2021, *13*, 1561.. Viruses.

[38] Koehler F.C., Di Cristanziano V., Späth M.R., Hoyer-Allo K.J.R., Wanken M., Müller R.-U., Burst V. (2022). The Kidney in Hantavirus Infection—Epidemiology, Virology, Pathophysiology, Clinical Presentation, Diagnosis and Management. Clin. Kidney J..

[39] Riccò M., Ferraro P., Peruzzi S., Balzarini F., Ranzieri S. (2021). Hantaviruses in Agricultural and Forestry Workers: Knowledge, Attitudes and Practices in Italian Physicians. Trop. Med. Infect. Dis..

[40] Li D., Schmaljohn A.L., Anderson K., Schmaljohn C.S. (1995). Complete Nucleotide Sequences of the M and S Segments of Two Hantavirus Isolates from California: Evidence for Reassortment in Nature among Viruses Related to Hantavirus Pulmonary Syndrome. Virology.

[41] Razzauti M., Plyusnina A., Henttonen H., Plyusnin A. (2008). Accumulation of Point Mutations and Reassortment of Genomic RNA Segments Are Involved in the Microevolution of Puumala Hantavirus in a Bank Vole (Myodes Glareolus) Population. J. Gen. Virol..

[42] Razzauti M., Plyusnina A., Sironen T., Henttonen H., Plyusnin A. (2009). Analysis of Puumala Hantavirus in a Bank Vole Population in Northern Finland: Evidence for Co-Circulation of Two Genetic Lineages and Frequent Reassortment between Strains. J. Gen. Virol..

[43] Klempa B. (2018). Reassortment Events in the Evolution of Hantaviruses. Virus Genes.

[44] Myhrman G. (1951). Nephropathia Epidemica a New Infectious Disease in Northern Scandinavia. J. Intern. Med..

[45] Smadel J.E. (1953). Epidemic Hemorrhagic Fever. Am. J. Public Health Nations Health.

[46] Powell G.M. (1954). Hemorrhagic Fever: A Study of 300 Cases. Medicine.

[47] Lähdevirta J. (1971). Nephropathia Epidemica in Finland. A Clinical Histological and Epidemiological Study. Ann. Clin. Res..

[48] Noh J.Y., Cheong H.J., Song J.Y., Kim W.J., Song K.-J., Klein T.A., Lee S.H., Yanagihara R., Song J.-W. (2013). Clinical and Molecular Epidemiological Features of Hemorrhagic Fever with Renal Syndrome in Korea over a 10-Year Period. J. Clin. Virol..

[49] Jiang H., Du H., Wang L.M., Wang P.Z., Bai X.F. (2016). Hemorrhagic Fever with Renal Syndrome: Pathogenesis and Clinical Picture. Front. Cell. Infect. Microbiol..

[50] Zou L.-X., Chen M.-J., Sun L. (2016). Haemorrhagic Fever with Renal Syndrome: Literature Review and Distribution Analysis in China. Int. J. Infect. Dis..

[51] Duchin J.S., Koster F.T., Peters C.J., Simpson G.L., Tempest B., Zaki S.R., Ksiazek T.G., Rollin P.E., Nichol S., Umland E.T. (1994). Hantavirus Pulmonary Syndrome: A Clinical Description of 17 Patients with a Newly Recognized Disease. N. Engl. J. Med..

[52] Ketai L.H., Williamson M.R., Telepak R.J., Levy H., Koster F.T., Nolte K.B., Allen S.E. (1994). Hantavirus Pulmonary Syndrome: Radiographic Findings in 16 Patients. Radiology.

[53] Llah S.T., Mir S., Sharif S., Khan S., Mir M.A. (2018). Hantavirus Induced Cardiopulmonary Syndrome: A Public Health Concern. J. Med. Virol..

[54] CDC (1993). Outbreak of Acute Illness--Southwestern United States, 1993. MMWR Morb Mortal Wkly Rep..

[55] Leopardi S., Drzewnioková P., Baggieri M., Marchi A., Bucci P., Bregoli M., De Benedictis P., Gobbo F., Bellinati L., Citterio C. (2022). Identification of Dobrava-Belgrade Virus in Apodemus Flavicollis from North-Eastern Italy during Enhanced Mortality. Viruses.

[56] Kim W.-K., No J.S., Lee D., Jung J., Park H., Yi Y., Kim J.-A., Lee S.-H., Kim Y., Park S. (2020). Active Targeted Surveillance to Identify Sites of Emergence of Hantavirus. Clin. Infect. Dis..

[57] Tortosa F., Perre F., Tognetti C., Lossetti L., Carrasco G., Guaresti G., Iglesias A., Espasandin Y., Izcovich A. (2024). Seroprevalence of Hantavirus Infection in Non-Epidemic Settings over Four Decades: A Systematic Review and Meta-Analysis. BMC Public Health.

[58] Acham-Roschitz B., Aberle S.W., Pirker N., Kaulfersch W., Boehm M., Roedl S., Zenz W., Ring E., Mache C.J. (2010). Nephropathia Epidemica (Puumala Virus Infection) in Austrian Children. Pediatr. Infect. Dis. J..

[59] Echterdiek F., Kitterer D., Alscher M.D., Schwenger V., Ruckenbrod B., Bald M., Latus J. (2019). Clinical Course of Hantavirus-Induced Nephropathia Epidemica in Children Compared to Adults in Germany—Analysis of 317 Patients. Pediatr. Nephrol..

[60] Gilson G.J., Maciulla J.A., Nevils B.G., Izquierdo L.E., Chatterjee M.S., Curet L.B. (1994). Hantavirus Pulmonary Syndrome Complicating Pregnancy. Am. J. Obstet. Gynecol..

[61] Hofmann J., Führer A., Bolz M., Waldschläger-Terpe J., Meier M., Lüdders D., Enders M., Oltmann A., Meisel H., Krüger D.H. (2012). Hantavirus Infections by Puumala or Dobrava-Belgrade Virus in Pregnant Women. J. Clin. Virol..

[62] Ji F., Zhao W., Liu H., Zheng H., Wang S., He C., Wang W., Zhang R., Bai D., Tian C. (2017). Hemorrhagic Fever with Renal Syndrome Caused by Hantaan Virus Infection in Four Pregnant Chinese Women. J. Med. Virol..

[63] Bellomo C., Alonso D., Coelho R., Iglesias A., Periolo N., Martínez V.P. (2020). A Newborn Infected by Andes Virus Suggests Novel Routes of Hantavirus Transmission: A Case Report. Clin. Microbiol. Infect..

[64] Ferrés M., Martínez-Valdebenito C., Angulo J., Henríquez C., Vera-Otárola J., Vergara M.J., Pérez J., Fernández J., Sotomayor V., Valdés M.F. (2020). Mother-to-Child Transmission of Andes Virus through Breast Milk, Chile1. Emerg. Infect. Dis..

[65] Yang X., Yu C., Chen Y., Nian B., Chai M., Maimaiti D., Xu D., Zang X. (2024). Hemorrhagic Fever with Renal Syndrome Complicated by Acute Pancreatitis, High Intraocular Pressure, and Pulmonary Involvement: A Case Report. Infect. Drug Resist..

[66] Geeraedts F., Wevers M., Bosma F., Boer M.D., Brinkman J.N., Delsing C., GeurtsvanKessel C., Rockx B., Van Der Zanden A., Laverman G.D. (2024). Use of a Diagnostic Puumala Virus Real-Time RT-PCR in an Orthohantavirus Endemic Region in the Netherlands. Microbiol. Spectr..

[67] Zhao H.-D., Sun J.-J., Liu H.-L. (2023). Potential Clinical Biomarkers in Monitoring the Severity of Hantaan Virus Infection. Cytokine.

[68] European Centre for Disease Prevention and Control (2018). Annual Epidemiological Report for 2016: Hantavirus Infection.

[69] Yanagihara R., Gu S.H., Arai S., Kang H.J., Song J.-W. (2014). Hantaviruses: Rediscovery and New Beginnings. Virus Res..

[70] CDC Clinician Brief: Hantavirus Pulmonary Syndrome (HPS). https://www.cdc.gov/hantavirus/hcp/clinical-overview/hps.html.

[71] CDC Reported Cases of Hantavirus Disease. https://www.cdc.gov/hantavirus/data-research/cases/index.html.

[72] European Centre for Disease Prevention and Control (2023). Hantavirus Infection Annual Epidemiological Report for 2020.

[73] Wang Y.X.G., Voutilainen L., Aminikhah M., Helle H., Huitu O., Laakkonen J., Lindén A., Niemimaa J., Sane J., Sironen T. (2023). The Impact of Wildlife and Environmental Factors on Hantavirus Infection in the Host and Its Translation into Human Risk. Proc. R. Soc. B.

[74] European Centre for Disease Prevention and Control Surveillance and Updates for Hantavirus. https://www.ecdc.europa.eu/en/hantavirus-infection/surveillance-and-disease-data.

[75] Mustonen J., Henttonen H., Vaheri A. (2024). Hantavirus Infections among Military Forces. Mil. Med..

[76] European Centre for Disease Prevention and Control Disease Information about Hantavirus. https://www.ecdc.europa.eu/en/hantavirus-infection/facts.

[77] Munir N., Jahangeer M., Hussain S., Mahmood Z., Ashiq M., Ehsan F., Akram M., Ali Shah S.M., Riaz M., Sana A. (2021). Hantavirus Diseases Pathophysiology, Their Diagnostic Strategies and Therapeutic Approaches: A Review. Clin. Exp. Pharmacol. Physiol..

[78] Escadafal C., Avšič-Županc T., Vapalahti O., Niklasson B., Teichmann A., Niedrig M., Donoso-Mantke O. (2012). Second External Quality Assurance Study for the Serological Diagnosis of Hantaviruses in Europe. PLoS Negl. Trop. Dis..

[79] Lederer S., Lattwein E., Hanke M., Sonnenberg K., Stoecker W., Lundkvist Å., Vaheri A., Vapalahti O., Chan P.K.S., Feldmann H. (2013). Indirect Immunofluorescence Assay for the Simultaneous Detection of Antibodies against Clinically Important Old and New World Hantaviruses. PLoS Negl. Trop. Dis..

[80] Li W., Cao S., Zhang Q., Li J., Zhang S., Wu W., Qu J., Li C., Liang M., Li D. (2017). Comparison of Serological Assays to Titrate Hantaan and Seoul Hantavirus-Specific Antibodies. Virol. J..

[81] Kramski M., Meisel H., Klempa B., Krüger D.H., Pauli G., Nitsche A. (2007). Detection and Typing of Human Pathogenic Hantaviruses by Real-Time Reverse Transcription-PCR and Pyrosequencing. Clin. Chem..

[82] Altona Diagnostics RealStar® Hantavirus-HFRS RT-PCR Kit 1.0 RUO. https://altona-diagnostics.com/product/realstar-hantavirus-hfrs-rt-pcr-kit-1-0-ruo/.

[83] Altona Diagnostics RealStar® Hantavirus-HPS RT-PCR Kit 1.0 RUO. https://altona-diagnostics.com/product/realstar-hantavirus-hps-rt-pcr-kit-1-0-ruo/.

[84] Nunes B.T.D., De Mendonça M.H.R., Simith D.D.B., Moraes A.F., Cardoso C.C., Prazeres I.T.E., De Aquino A.A., Santos A.D.C.M., Queiroz A.L.N., Rodrigues D.S.G. (2019). Development of RT-qPCR and Semi-Nested RT-PCR Assays for Molecular Diagnosis of Hantavirus Pulmonary Syndrome. PLoS Negl. Trop. Dis..

[85] Weidmann M., Schmidt P., Vackova M., Krivanec K., Munclinger P., Hufert F.T. (2005). Identification of Genetic Evidence for Dobrava Virus Spillover in Rodents by Nested Reverse Transcription (RT)-PCR and TaqMan RT-PCR. J. Clin. Microbiol..

[86] Niskanen S., Jääskeläinen A., Vapalahti O., Sironen T. (2019). Evaluation of Real-Time RT-PCR for Diagnostic Use in Detection of Puumala Virus. Viruses.

[87] Aitichou M., Saleh S.S., McElroy A.K., Schmaljohn C., Ibrahim M.S. (2005). Identification of Dobrava, Hantaan, Seoul, and Puumala Viruses by One-Step Real-Time RT-PCR. J. Virol. Methods.

[88] Pang Z., Li A., Li J., Qu J., He C., Zhang S., Li C., Zhang Q., Liang M., Li D. (2014). Comprehensive Multiplex One-Step Real-Time TaqMan qRT-PCR Assays for Detection and Quantification of Hemorrhagic Fever Viruses. PLoS ONE.

[89] Klempa B., Fichet-Calvet E., Lecompte E., Auste B., Aniskin V., Meisel H., Denys C., Koivogui L., ter Meulen J., Krüger D.H. (2006). Hantavirus in African Wood Mouse, Guinea. Emerg. Infect. Dis..

[90] Erdin M., Stanoeva K.R., Mögling R., Korva M., Knap N., Resman Rus K., Domingo C., Reimerink J.H., De Vries A., Alburkat H. (2023). External Quality Assessment of Orthohantavirus and Lymphocytic Choriomeningitis Virus Molecular Detection and Serology in Europe, 2021. Eurosurveillance.

[91] Bagamian K.H., Towner J.S., Kuenzi A.J., Douglass R.J., Rollin P.E., Waller L.A., Mills J.N. (2012). Transmission Ecology of Sin Nombre Hantavirus in Naturally Infected North American Deermouse Populations in Outdoor Enclosures. PLoS ONE.

[92] Smith C.S., Underwood D.J., Gordon A., Pyne M.J., Smyth A., Genge B., Driver L., Mayer D.G., Oakey J. (2025). Identification and Epidemiological Analysis of a Putative Novel Hantavirus in Australian Flying Foxes. Virus Genes.

[93] Zhang Y.-Z., Zhang F.-X., Gao N., Wang J.-B., Zhao Z.-W., Li M.-H., Chen H.-X., Zou Y., Plyusnin A. (2009). Hantaviruses in Rodents and Humans, Inner Mongolia Autonomous Region, China. Emerg. Infect. Dis..

[94] Plyusnin A., Hörling J., Kanerva M., Mustonen J., Cheng Y., Partanen J., Vapalahti O., Kukkonen S.K., Niemimaa J., Henttonen H. (1997). Puumala Hantavirus Genome in Patients with Nephropathia Epidemica: Correlation of PCR Positivity with HLA Haplotype and Link to Viral Sequences in Local Rodents. J. Clin. Microbiol..

[95] Puthavathana P., Ho W.L., Yong Kang C. (1992). Typing of *Hantaviruses* from Five Continents by Polymerase Chain Reaction. Virus Res..

[96] Nordström H., Johansson P., Li Q., Lundkvist Å., Nilsson P., Elgh F. (2004). Microarray Technology for Identification and Distinction of Hantaviruses. J. Med. Virol..

[97] Filippone C., Castel G., Murri S., Ermonval M., Korva M., Avšič-Županc T., Sironen T., Vapalahati O., McElhinney L.M., Ulrich R.G. (2019). Revisiting the Genetic Diversity of Emerging Hantaviruses Circulating in Europe Using a Pan-Viral Resequencing Microarray. Sci. Rep..

[98] Oude Munnink B.B., Münger E., Nieuwenhuijse D.F., Kohl R., Van Der Linden A., Schapendonk C.M.E., Van Der Jeugd H., Kik M., Rijks J.M., Reusken C.B.E.M. (2020). Genomic Monitoring to Understand the Emergence and Spread of Usutu Virus in the Netherlands, 2016–2018. Sci. Rep..

[99] Gire S.K., Goba A., Andersen K.G., Sealfon R.S.G., Park D.J., Kanneh L., Jalloh S., Momoh M., Fullah M., Dudas G. (2014). Genomic Surveillance Elucidates Ebola Virus Origin and Transmission during the 2014 Outbreak. Science.

[100] Kim W.-K., Cho S., Lee S.-H., No J.S., Lee G.-Y., Park K., Lee D., Jeong S.T., Song J.-W. (2021). Genomic Epidemiology and Active Surveillance to Investigate Outbreaks of Hantaviruses. Front. Cell. Infect. Microbiol..

[101] Klein T.A., Kim H.-C., Chong S.-T., Kim J.-A., Lee S.-Y., Kim W.-K., Nunn P.V., Song J.-W. (2015). Hantaan Virus Surveillance Targeting Small Mammals at Nightmare Range, a High Elevation Military Training Area, Gyeonggi Province, Republic of Korea. PLoS ONE.

[102] Hjelle B., Torrez-Martinez N., Koster F.T., Jay M., Ascher M.S., Brown T., Reynolds P., Ettestad P., Voorhees R.E., Sarisky J. (1996). Epidemiologic Linkage of Rodent and Human Hantavirus Genomic Sequences in Case Investigations of Hantavirus Pulmonary Syndrome. J. Infect. Dis..

[103] Schilling S., Emmerich P., Klempa B., Auste B., Schnaith E., Schmitz H., Krüger D.H., Günther S., Meisel H. (2007). Hantavirus Disease Outbreak in Germany: Limitations of Routine Serological Diagnostics and Clustering of Virus Sequences of Human and Rodent Origin. J. Clin. Microbiol..

[104] Quer J., Colomer-Castell S., Campos C., Andrés C., Piñana M., Cortese M.F., González-Sánchez A., Garcia-Cehic D., Ibáñez M., Pumarola T. (2022). Next-Generation Sequencing for Confronting Virus Pandemics. Viruses.

[105] Carroll M.W., Matthews D.A., Hiscox J.A., Elmore M.J., Pollakis G., Rambaut A., Hewson R., García-Dorival I., Bore J.A., Koundouno R. (2015). Temporal and Spatial Analysis of the 2014–2015 Ebola Virus Outbreak in West Africa. Nature.

[106] Siddle K.J., Eromon P., Barnes K.G., Mehta S., Oguzie J.U., Odia I., Schaffner S.F., Winnicki S.M., Shah R.R., Qu J. (2018). Genomic Analysis of Lassa Virus during an Increase in Cases in Nigeria in 2018. N. Engl. J. Med..

[107] Grubaugh N.D., Ladner J.T., Kraemer M.U.G., Dudas G., Tan A.L., Gangavarapu K., Wiley M.R., White S., Thézé J., Magnani D.M. (2017). Genomic Epidemiology Reveals Multiple Introductions of Zika Virus into the United States. Nature.

[108] No J.S., Kim W.-K., Cho S., Lee S.-H., Kim J.-A., Lee D., Song D.H., Gu S.H., Jeong S.T., Wiley M.R. (2019). Comparison of Targeted Next-Generation Sequencing for Whole-Genome Sequencing of Hantaan Orthohantavirus in Apodemus Agrarius Lung Tissues. Sci. Rep..

[109] McMullan L.K., Albariño C.G., Ksiazek T.G., Nichol S.T., Spiropoulou C.F. (2018). Complete Genome Sequences of a Hantavirus Isolate from New York. Genome Announc..

[110] Cho S., Kim W.-K., No J.S., Lee S.-H., Jung J., Yi Y., Park H.C., Lee G.-Y., Park K., Kim J.-A. (2021). Urinary Genome Detection and Tracking of Hantaan Virus from Hemorrhagic Fever with Renal Syndrome Patients Using Multiplex PCR-Based next-Generation Sequencing. PLoS Negl. Trop. Dis..

[111] Pérez-Umphrey A.A., Settlecowski A.E., Elbers J.P., Williams S.T., Jonsson C.B., Bonisoli-Alquati A., Snider A.M., Taylor S.S. (2023). Genetic Variants Associated with Hantavirus Infection in a Reservoir Host Are Related to Regulation of Inflammation and Immune Surveillance. Infect. Genet. Evol..

[112] Taylor M.K., Williams E.P., Wongsurawat T., Jenjaroenpun P., Nookaew I., Jonsson C.B. (2020). Amplicon-Based, Next-Generation Sequencing Approaches to Characterize Single Nucleotide Polymorphisms of Orthohantavirus Species. Front. Cell. Infect. Microbiol..

[113] Park K., Lee S.-H., Kim J., Lee J., Lee G.-Y., Cho S., Lee S.H., Park K., No J.S., Budhathoki S. (2021). Multiplex PCR-Based Nanopore Sequencing and Epidemiological Surveillance of Hantaan Orthohantavirus in Apodemus Agrarius, Republic of Korea. Viruses.

[114] Wichmann D., Slenczka W., Alter P., Boehm S., Feldmann H. (2001). Hemorrhagic Fever with Renal Syndrome: Diagnostic Problems with a Known Disease. J. Clin. Microbiol..

[115] Galeno H., Mora J., Villagra E., Fernandez J., Hernandez J., Mertz G.J., Ramirez E. (2002). First Human Isolate of Hantavirus (*Andes Virus*) in the Americas. Emerg. Infect. Dis..

[116] Kruger D.H., Figueiredo L.T.M., Song J.-W., Klempa B. (2015). Hantaviruses—Globally Emerging Pathogens. J. Clin. Virol..

[117] Hantavirus Outbreak Toolbox. https://www.who.int/emergencies/outbreak-toolkit/disease-outbreak-toolboxes/hantavirus-outbreak-toolbox.

[118] PAHO Strengthens Capacities for Hantavirus and Arenavirus Surveillance in the Americas. https://www.paho.org/en/news/13-3-2024-paho-strengthens-capacities-hantavirus-and-arenavirus-surveillance-americas#:~:text=This%20international%20collaboration%20will%20not,fight%20against%20emerging%20and%20re%2D.

[119] Knust B., Rollin P.E. (2013). Twenty-Year Summary of Surveillance for Human Hantavirus Infections, United States. Emerg. Infect. Dis..

[120] Hantaviruses (Infection with). https://www.woah.org/app/uploads/2022/02/hantaviruses-infection-with.pdf.

